# Fluorescence Imaging of Extracellular Potassium Ion Using Potassium Sensing Oligonucleotide

**DOI:** 10.3389/fchem.2022.922094

**Published:** 2022-07-08

**Authors:** Shinobu Sato, Shinsuke Ohzawa, Kojiro Sota, Naoto Sakamoto, Ayano Udo, Shinji Sueda, Tomoki Matsuda, Takeharu Nagai, Shigeori Takenaka

**Affiliations:** ^1^ Department of Applied Chemistry, Kyushu Institute of Technology, Kitakyushu, Japan; ^2^ Department of Bioscience and Bioinformatics, Kyushu Institute of Technology, Iizuka, Japan; ^3^ SANKEN (The Institute of Scientific and Industrial Research), Osaka University, Suita, Japan

**Keywords:** potassium ion, sodium ion, G-quadruplex, fluorometric imaging, cell surface, potassium sensing oligonucleotide, potassium ion efflux

## Abstract

Potassium-sensing oligonucleotide, PSO, a conjugate of a quadruplex structure-forming oligonucleotide with a peptide incorporating a Förster Resonance Energy Transfer (FRET) chromophore pair, has been developed for fluorescent detection of potassium ion (K^+^) in aqueous medium. PSO **1** could be introduced into cells for real-time imaging of cytoplasmic K^+^ concentrations. To perform fluorescent imaging of K^+^ on the cell surface, we synthesized twelve PSO derivatives with different types of peptide types and lengths, and oligonucleotide sequences including thrombin-binding aptamer (TBA) sequences with FAM and TAMRA as a FRET chromophore pair, and evaluated their performance. **1** was shown to respond selectively to K^+^, not to most ions present *in vivo*, and to show reciprocal fluorescence changes in response to K^+^ concentration. For the peptide chains and oligonucleotide sequences examined in this study, the PSO derivatives had *K*_d_ values for K^+^ in the range of 5–30 mM. All PSO derivatives showed high K^+^ selectivity even in the presence of excess Na^+^. The PSO derivatives were successfully localized to the cell surface by biotinylated concanavalin A (ConA) or sulfo-NHS-biotin *via* streptavidin (StAv). Fluorescence imaging of extracellular K^+^ upon addition of apoptosis inducers was successfully achieved by **1** localized to the cell surface.

## 1 Introduction

Many metal ions are present in living organisms and play important roles in biological activities. Potassium ion, K^+^, the most abundant metal cation in cells, is responsible for maintaining cell membrane potential and is involved in vital phenomena such as neurotransmission, cardiac excitability, epithelial fluid transport, muscle contraction, and cell proliferation (Shieh et al., 2000). Thus, abnormal K^+^ concentrations *in vivo* cause several diseases such as hypertension, heart disease, seizures, or strokes (Viera et al., 2005). From this perspective, monitoring of K^+^ *in vivo* has become necessary. Blood K^+^ concentrations are measured by blood tests using ion-selective electrodes (Fogh-Andersen et al., 1984). Microelectrode-based techniques have been used to monitor intracellular and extracellular K^+^. However, the use of microelectrodes at the single-cell level is not only technically difficult, but only local sites can be analyzed (Messerli et al., 2009). Fluorescent K^+^ probes have been developed, and azacrown ether-type PBFI is commercially available, but it has the problem of low selectivity of K^+^ for Na^+^ because it also responds to Na^+^ (Meuwis et al., 1995). TAC-Red, a triazacryptant (TAC) type, has shown high K^+^ selectivity (Padmawar et al., 2005). GEPIIs have also been developed by using recombinant protein technology (Bischof et al., 2017). These probes are mainly excellent for real-time monitoring of intracellular K^+^ concentration changes. However, fluorescent imaging reagents for K^+^ at the cell surface have not been thoroughly studied. Urano and co-workers (Hirata et al., 2018) have therefore developed TSLHalo, which can immobilize TAC via halo tags on membrane proteins.

Takenaka’s group developed a probe for fluorescent imaging of K^+^ concentrations in homogeneous aqueous solutions by introducing Förster Resonance Energy Transfer (FRET) dye pairs at both ends of DNA with a quadruplex DNA structure-forming sequence, which they named potassium-sensing oligonucleotide (PSO) (Takenaka, 2021). The four-stranded structure is known to be K^+^ stabilized, and K^+^ forms a quadruplex structure. The intensity of the FRET signal is expected to correlate with the K^+^ concentration. Juskowiak and co-workers (Dembska et al., 2020) have successfully localized the cell surface by conjugating cholesterol. Takenaka’s group further introduced biotin into PSO to form a complex with avidin, which was then introduced into cells and successfully monitored changes in intracellular K^+^ concentration in real time (Ohtsuka et al., 2012).

**FIGURE 1:**
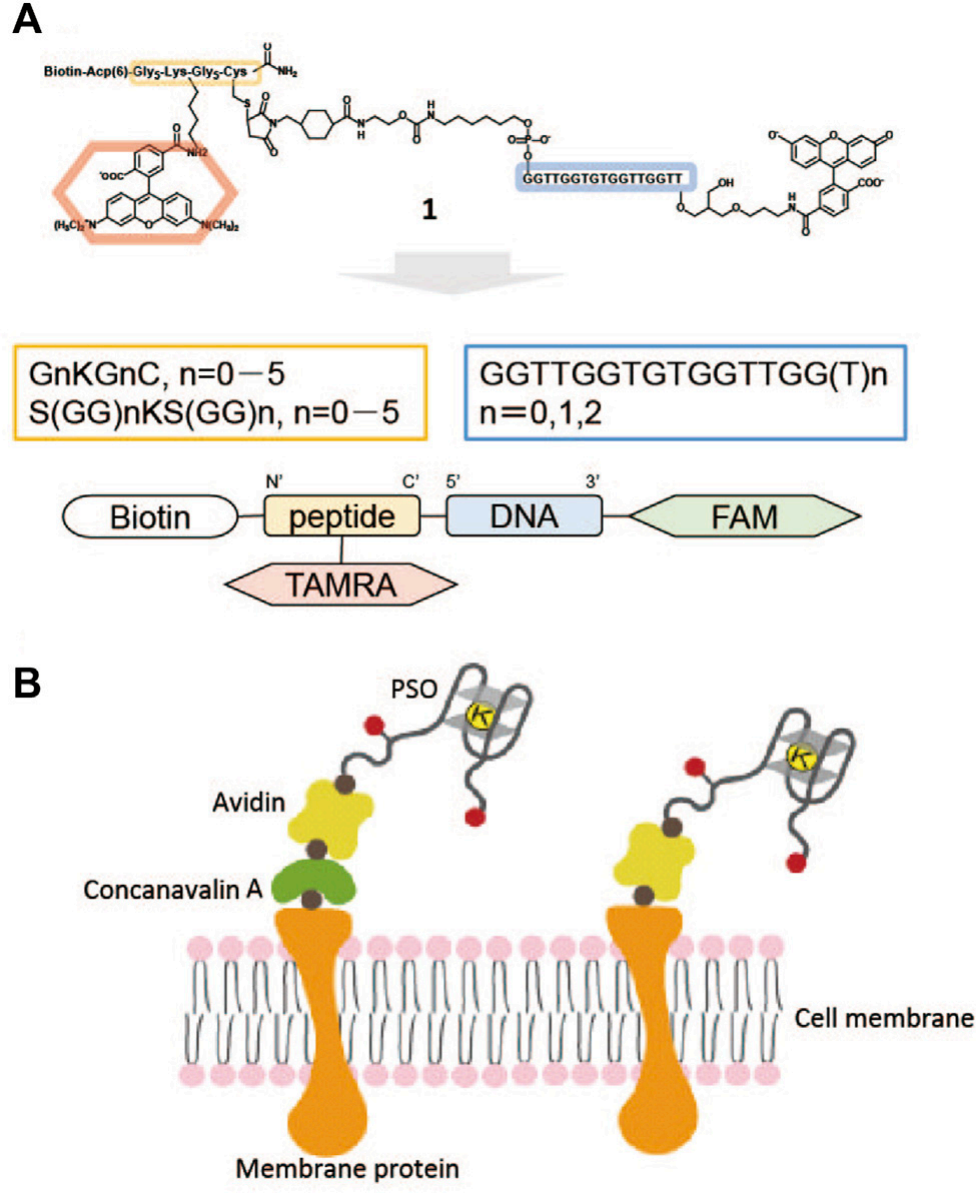
**(A)** Example of potassium-sensing oligonucleotide (PSO) **1** consisting of oligonucleotide-carrying TBA sequence, peptide linker with biotin, and FRET chromophore pair of FAM and TAMRA. **(B)** PSO localized on a cell surface through sugar chain, concanavalin A (ConA), and avidin (left) or biotinylated membrane protein, avidin (right). Black circle: biotin; red circle: FRET chromophore pair.

Here, peptide sequences, oligonucleotide sequences were examined to improve PSO performance (Figure 1A). We also attempted extracellular display of PSO and extracellular K^+^ imaging (Figure 1B).

## 2 Experimental

### 2.1 Materials

PSO **1** as a peptide-oligonucleotide conjugate used in this article was synthesized according to the previous report (Ohtsuka et al., 2012). **2**–**12** used in this article were synthesized according to the procedure of **1** (Supplementary Figures S1–S3).

### 2.2 Fluorescence Measurement

Seven hundred μl volume of 0.2 μM **1** (or **2**–**12**) in 20 mM Tris-HCl buffer (pH 7.4) in the presence or absence of 0.3 μM streptavidin (StAv) was placed in a quartz cell (1 cm light path) and fluorescence spectra were measured in the 500–700 nm spectral range (excitation wavelength at 495 nm, PMV = 650 V, and 10 nm slits) by using the F-7000 Fluorescence Spectrometer (HITACHI) upon addition of MilliQ water containing KCl, NaCl, MgCl_2_, CaCl_2_, CH_3_COONH_4_, or LiCl and subsequently mixing by moving the cell up and down ten times and keeping 3 min at 25°C.

The oligonucleotide sequence, TBA, formed the tetraplex structure with two G-quartet plates with 1:1 complex with K^+^ or Na^+^. Spectra changes of fluorescence titration upon addition of K^+^ or Na^+^ was consistent with the following Eq. 1 to detect the dissociation constant, *K*_d_/M, where Φ_0_ and Φ_i_ refer to quantum yield of the chromophore part in the absence and presence of metal cations (M^+^), ε_0_ or ε_i_ also refers to molar absorptivity of the chromophore part in the absence or presence of M^+^, and R_0_ or R_i_ also refers to fluorescence ratio (F_585_/F_517_ at Ex = 495 nm) of the chromophore part in the absence or presence of M^+^ (Ohtsuka et al., 2012):

$$\frac{\left(R_{0}-R_{i}\right)}{R_{0}}=\frac{\varepsilon_{0}\Phi_{0}-\varepsilon_{i}\Phi_{i}}{\varepsilon_{0}\Phi_{0}}\times \frac{\left[M^{+}\right]}{K_{d}+\left[M^{+}\right]}$$ (1)

### 2.3 Fluorescence Imaging of 1 Immobilized on the Cell

The immobilization procedure using concanavalin A (ConA) was carried out according to the previously published report (Sato et al., 2019). HeLa cell was cultivated in a collagen-cored glass bottom dish in DMEM medium. This medium was removed and washed with 1 ml of 1×PBS two times. Two-hundred microliter of 0.5 μM ConA biotin conjugate (Type IV, SIGMA-ALDRICH, 4–8 mol biotin per mol protein) in DMEM (+10% FBS) was added in this dish and incubated for 10 min at 37°C under 5% CO_2_. After this solution was removed and washed with 1 ml of 1×PBS, 100 μl of 5.0 µM StAv in DMEM (+10% FBS) was added in this dish and incubated for 10 min at 37°C under 5% CO_2_. Finally, 100 μl of 5.0 µM **1** in DMEM (+10% FBS) was added and incubated for 10 min at 37°C under 5% CO_2_ after washing with 1 ml of 1×PBS. After this solution was removed and washed with 1 ml of 1×PBS, 2 ml of DMEM (+10% FBS) was added in this dish and confocal laser fluorescence inverted microscopy was carried out using FV1000 (Olympus). Oil immersion lens at 60-fold magnification (UPIanSApo ×60/1.40 oil) was used as an object glass and the CCD camera imaging was carried out on an incubation stage for living cells at 37°C under 5% CO_2_ atmosphere. Imaging conditions; *λ*_ex_: 488 nm, Ch1: HV = 660, Gain = 1×, offset = 0%, *λ* = 508–528 nm (SDM560), Ch2: HV = 660, Gain = 1×, offset = 0%, *λ* = 575–595 nm (mirror), Size: 516 × 516, Kalmen: 2.

The immobilization procedure using EZ-Link™ Sulfo-NHS-biotin (Thermo) is as follows. HeLa cell was cultivated in collagen-cored glass bottom dish in DMEM medium. This medium was removed and washed with 2 ml of 1×PBS two times. One-hundred microliter of 0.5 mg/ml sulfo-NHS-Biotin in 1×PBS was added in this dish and incubated for 10 min at 15°C. After removal, this solution was washed with 2 ml of 1×PBS twice, 70 μl of 5.0 µM StAv in DMEM (+10% FBS) was added in this dish and incubated for 10 min at 37°C under 5% CO_2_. Finally, 70 μl of 5.0 µM **1** in DMEM (+10% FBS) was added and incubated for 10 min at 37°C under 5% CO_2_ after washing with 1 ml of 1×PBS. After removal, this solution was washed with 1 ml of 1×PBS, 2 ml of DMEM (+10% FBS) was added in this dish. Imaging using Confocal Microscopy System A1 (Nikon) was performed with each addition of KCl. Imaging conditions; Oil immersion lens: 60×/1.40 oil apo, *λ*_ex_: 488 nm, Ch: 500–554 nm for FAM, 554–620 nm for TAMRA, Laser intensity; 2.0, Gain = 145, Size: 516 × 516, Scan number: 4.

### 2.4 Fluorescence Imaging of 1 Immobilized on the Cell After Adding Apoptosis Inducer

The immobilization procedure using ConA was carried out according to the same procedure shown in 2.3. Fluorescent images were taken every 3 min from the start of observation using FV1000. Twelve minutes after the start of observation, Amphotericin B (SIGMA-ALDRICH), Oubain (BIOMOL GmbH, Hamburg, Germany), and Bumetanide (SIGMA-ALDRICH) (final conc. 10 μM each) were added, and the images were continuously taken every 3 min. Imaging conditions; *λ*_ex_ = 488 nm, Ch1: HV = 660, Gain = 1×, offset = 0%, *λ* = 508–528 nm (SDM560), Ch2: HV = 660, Gain = 1×, offset = 0%, *λ* = 575–595 nm (mirror), Size: 256 × 256, Kalmen: 2.

The immobilization procedure using EZ-Link™ Sulfo-NHS-biotin was carried out according to the same procedure shown in 2.3. Fluorescent images were taken every 1 min from the start of observation by using ECLIPSE Ti. Ten minutes after the start of observation, Amphotericin B (final conc. 26 μM) was added, and the images were continuously taken every 1 min. Imaging conditions; Oil immersion lens: 60×/1.40 oil apo, *λ*_ex_: 488 nm, Ch: 500–554 nm for FAM, 554–620 nm for TAMRA, Laser intensity; 2.0, Gain = 200, Size: 516 × 516, Scan number: 4.

## 3 Result and Discussion

### 3.1 Design of PSO Derivatives

PSO was firstly synthesized by Takenaka et al. (Ueyama et al., 2002) by introducing FAM and TAMREA at both ends of oligonucleotides bearing human telomere sequences. The FRET signal of this PSO derivative changed with K^+^ concentration. Na^+^ and other divalent cations present *in vivo* did not affect the change in PSO fluorescence with changing K^+^ concentrations. Subsequent studies on human telomere DNA have revealed that there is diversity in the G-quartet DNA structure formed by the type of coexisting cation (Dai et al., 2008). Therefore, Takenaka’s group has investigated the use of thrombin-binding aptamer (TBA) sequences as a substitute for human telomere sequences to eliminate heterogeneity to the G-quadruplex structure (Nagatoishi et al., 2006). However, when the FRET chromophore pair was introduced directly into the TBA sequence, it was found that the two chromophores were too close to each other, which increased FRET efficiency but also caused quenching at the same time. Therefore, oligonucleotide chains were introduced at the ends of the TBA sequences to change the distance between the chromophores. This sequence modification increased the FRET efficiency in this PSO derivative but decreased the dissociation constant for K^+^. This was thought to be due to the introduction of a polyanion at the PSO terminus that is unrelated to the formation of the G-quartet structure. Therefore, by introducing a charge-free peptide sequence into PSO, Takenaka’s group succeeded in synthesizing a PSO derivative, **1**, without a decrease in the dissociation constant (Ohtsuka et al., 2012). Biotin was introduced in **1**. **1** was successfully retained in the cytoplasm by being made into an avidin complex together with a biotinylated nuclear efflux peptide, allowing real-time monitoring of K^+^ efflux from the cell during apoptosis. **1** should be localized to the cell surface by complex formation with biotin. Here we examined the localization of PSO on the cell surface and the effects of peptide sequence and length, as well as the effects of the terminal sequence of oligonucleotides with TBA sequences. Figure 1A summarizes the structural units of the PSO derivatives and how the units were modified for each. Specific structural unit combinations are summarized in Table 1 with altered peptide and oligonucleotide sequences.

**TABLE 1:** Effect of peptide and oligonucleotide portions within PSO on K^+^/Na^+^ selectivity.

PSO	Biotin- [Peptide] -C	DNA	KCl		NaCl		KCl (in 145 mM NaCl)		K^+^/Na^+^
			Kd/mM	ΔRatio	Kd/mM	ΔRatio	Kd/mM	ΔRatio	
1	GGGGG K GGGGG	GGTTGGTGTGGTTGGTT	21.2 ± 0.7	1.43	639 ± 24	0.52	17.9 ± 0.2	2.43	30
2	GGGGG K GGGGG	GGTTGGTGTGGTTGG	13.4 ± 0.6	2.77	677 ± 32	0.37	10.8 ± 0.4	2.34	50
3	GGGGG K GGG	GGTTGGTGTGGTTGG	9.4 ± 0.5	2.28	692 ± 39	0.63	8.9 ± 0.3	2.02	74
4	GGGGG K G	GGTTGGTGTGGTTGG	12.3 ± 0.6	2.76	538 ± 26	0.32	8.8 ± 0.2	2.49	44
5	GGGGG K	GGTTGGTGTGGTTGG	10.1 ± 0.5	3.05	564 ± 26	0.36	10.7 ± 0.4	2.75	56
6	GGSGG K GGSGG	GGTTGGTGTGGTTGGTT	29.8 ± 0.7	1.81	786 ± 24	0.22	23.7 ± 1.1	1.66	26
7	SGG K SGG	GGTTGGTGTGGTTGGTT	29.1 ± 0.8	1.66	802 ± 23	0.21	13.8 ± 0.3	1.66	28
8	GGG K GGG	GGTTGGTGTGGTTGGTT	22.0 ± 1.2	1.66	638 ± 18	0.22	17.7 ± 0.3	1.50	29
9	GGG K GGGGG	GGTTGGTGTGGTTGGTT	29.3 ± 1.7	1.71	930 ± 35	0.20	18.8 ± 0.4	1.52	32
10	(SGG)_5_ K S	GGTTGGTGTGGTTGG	5.1 ± 0.2	1.73	291 ± 17	0.29	5.1 ± 0.1	1.48	57
11	(SGG)_5_ K S	GGTTGGTGTGGTTGGT	15.7 ± 0.8	1.64	489 ± 11	0.23	14.0 ± 0.4	1.44	31
12	(SGG)_5_ K S	GGTTGGTGTGGTTGGTT	23.1 ± 0.6	1.00	561 ± 1	0.16	14.6 ± 0.2	1.48	24

ΔRatio: F_585_/F_517 at 150 mM KCl or NaCl_ - F_585_/F_517 at 0 mM KCl or NaCl、_ Conditions: 0–150 mM KCl (in 145 mM KCl) or 0–150 mM NaCl, 20 mM Tris-HCl (pH 7.4).

### 3.2 Ion Selectivity of PSO 1 in an Aqueous Medium

PSO alone is thought to form an elongated structure of oligonucleotides in aqueous solution, and in the presence of K^+^, PSO is expected to incorporate this to form a quadruplex structure. In this case, the FRET efficiency is expected to increase as the distance between the FAM and TAMRA in the PSO becomes closer. To demonstrate this, we evaluated ΔRatio, the ratio of fluorescence intensity at 585–517 nm. FRET of **1** responses was evaluated for various ionic species present *in vivo*.

**FIGURE 2:**
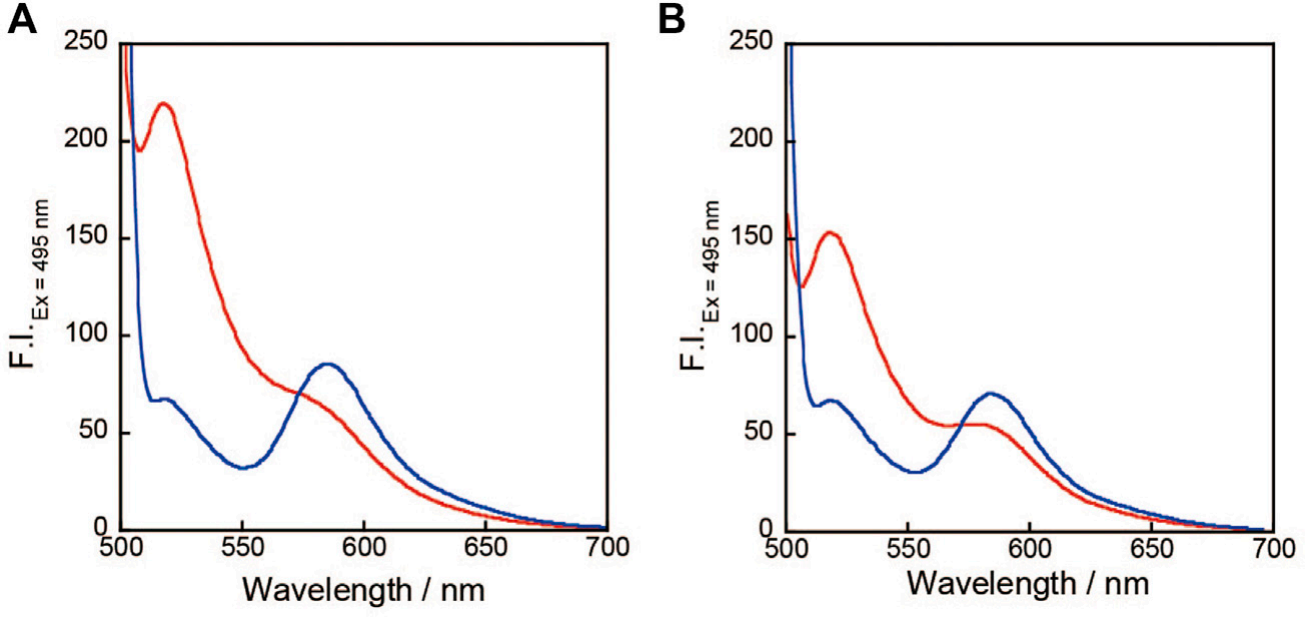
**(A)** Fluorescence spectra of 0.2 μM **1**, 0.3 μM StAv in the absence (red) or presence (blue) of 150 mM KCl, 20 mM Tris-HCl (pH 7.4) without **(A)** or with 145 mM NaCl **(B)**.

Considering that PSO localizes to the cell surface via StAv, as shown in Figure 1B, we measured the fluorescence change upon addition of K^+^ of StAv bound **1**. Upon addition of K^+^, the fluorescence of FAM decreased and that of TAMRA increased. Figure 2 shows the fluorescence spectra of 0.2 μM **1** bound to StAv only and in the presence of 150 mM K^+^ at an excitation wavelength of 495 nm. In the absence of K^+^, the fluorescence spectrum of **1** showed fluorescence of TAMRA in addition to that of FAM. The TAMRA fluorescence seen in the fluorescence spectrum of **1** only was smaller than that of **1** bound to StAv (Ohtsuka et al., 2012). This was thought to be due to the binding of **1** to high molecular weight StAv, which reduced the motility of **1**. Fluorescence changes of **1** in the presence of 145 mM NaCl, an extracellular condition, are shown in Figure 2B. Before the addition of KCl, FAM fluorescence was already suppressed and TAMRA fluorescence increased. This may be because the presence of excess Na^+^ neutralized the charge of the polyphosphate anion of oligonucleotide **1** and brought FAM and TAMRA into close proximity even in the absence of K^+^-induced quadruplex formation. Although this reduced the ratio range, the quantitative ratios changed with the addition of KCl. Furthermore, the fluorescence behavior of **1** at different pH environments from pH 7.4 was observed. The pH 3.6 was not measurable because no fluorescence of FAM was observed. The pH 6.4 showed a decrease in fluorescence of FAM and an increase in fluorescence of TAMRA with the addition of KCl, *K*_d_ = 12.5 ± 0.4 mM (ΔRatio 3.51). The p*K*_a_ of FAM was 6.4, and the fluorescence was quenched at more acidic pH 3.6, so the fluorescence could not be measured. The p*K*_a_ of FAM is 6.4, and the fluorescence was not quenched at pH 7.4–6.4, so the measurement could be performed at pH 7.4.

**FIGURE 3:**
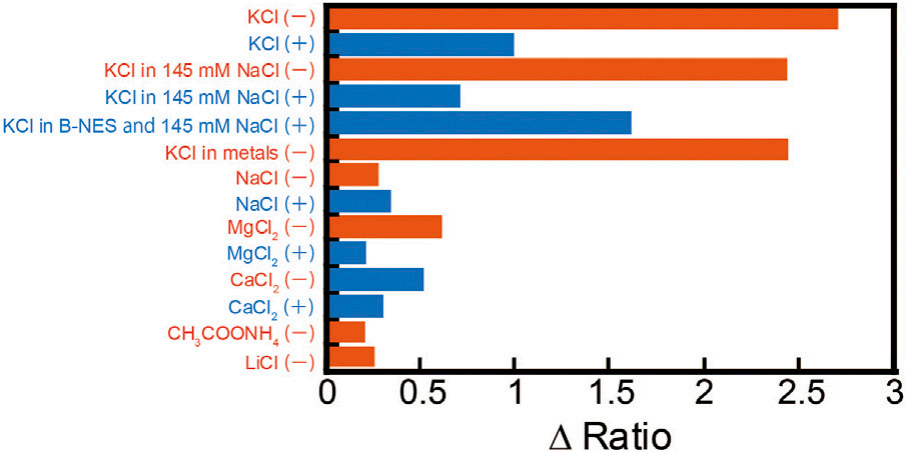
ΔRatio of 0.2 μM **1** in the absence (−) or presence (+) of 0.3 μM streptavidin (StAv) in 20 mM Tris-HCl (pH 7.4) under several cations; 150 mM KCl, 145 mM NaCl, 2 mM MgCl_2_, 2 mM CaCl_2_, 20 mM CH_3_COONH_4_, or 150 mM LiCl. Ex: 495 nm.

The ΔRatio was evaluated for 0.2 μM **1** with 150 mM KCl, 145 mM NaCl, 2 mM MgCl_2_, 2 mM CaCl_2_, 20 mM CH_3_COONH_4_, and 150 mM LiCl, respectively, or with all ions added. The ΔRatio of **1** when each metal ion is added and when all these ions are included is summarized in Figure 3. **1** showed a large change in ΔRatio from the decrease in FAM fluorescence and increase in TAMRA only in the case of KCl addition (Figure 3). This may be due to the fact that the TBA sequence site in **1** forms a quadruplex DNA structure only in the presence of K^+^. Furthermore, when other ions are included, there is almost no change in the ΔRatio, indicating that even when multiple ions are present inside and outside the cell, the ΔRatio changes significantly only when KCl is added. In the absence of 145 mM NaCl (Figure 2A), before the addition of KCl, **1** had a large fluorescence of FAM and a sufficient suppression of TAMRA fluorescence, and with the addition of KCl, a decrease in FAM fluorescence and an increase in TAMRA fluorescence were observed.

When KCl was added to 0.2 μM **1** in the presence of 0.3 μM StAv in 145 mM NaCl, the ΔRatio went from 2.4 in the absence of StAv to 0.7 (70% decrease). This ΔRatio recovered to 1.6 in the presence of biotinyl-nuclear export signal peptide (B-NES) and StAv. **1** to StAv decreased the range of ΔRatio not only in the case of KCl but also in the cases of NaCl, MgCl_2_, and CaCl_2_. The mixing ratio of StAv to **1** is 1:1, leaving three biotin binding sites in StAv. The recovery of ΔRatio was observed when B-NES was added to fill this empty binding site, suggesting that this phenomenon is caused by the effect on the empty binding site of StAv, resulting in a decrease in ΔRatio.

### 3.3 Examination of Peptide Linkers and Oligonucleotide Sequences in PSO

K^+^ selectivity and fluorescence behavior were investigated by examining the sequence and length of the peptide moiety and the sequence of the oligonucleotide moiety containing the TBA sequence introduced into the PSO. PSO **1** has GGGGGG K GGGGG as the peptide linker, with TAMRA modified at its central lysine (K). The length of the peptide linker is expected to affect the FRET efficiency between FAM and TAMRA at the oligonucleotide ends. **1** also has a FAM modified at the 3′-end of the TBA sequence part, but two thymines were inserted between them to inhibit quenching by the guanine at the 3′-end of the TBA sequence. Fluorescence spectra of 0.2 μM **1** (or **2**–**12**) in 20 mM Tris-HCl (pH 7.4) were confirmed in addition of KCl and with NaCl. *K*_d_ (NaCl)/*K*_d_ (KCl) from the *K*_d_ obtained from these fluorescence changes is shown as K^+^/Na^+^. To determine the selectivity of K^+^ under excess of Na^+^, KCl was added in the solution of **1** in the presence of 145 mM NaCl, and *K*_d_ for K^+^ was calculated and these results are shown in Table 1. All PSO derivatives showed selectivity for K^+^. Of particular interest is that the *K*_d_ for K^+^ in the presence of 145 mM NaCl was slightly lower than the *K*_d_ in the absence of NaCl. Although the exact reason for this is not clear, it is known that the volume of DNA shrinks at high salt concentrations (Chen et al., 2012), which may be due to the fact that K^+^-induced G4 structure formation is more likely to occur. As shown in Figure 3, the overall fluorescence intensity was lower in the presence of 145 mM NaCl, supporting this result. PSO derivatives, **2**, **3**, **4**, and **5** have 5, 3, 1, and 0 glycines linked from the 5′ end of the oligonucleotide, respectively. The ΔRatio values associated with the addition of KCl increased as the number of glycine was increased to 3, 1, and 0. Reciprocal fluorescence changes were observed as the distance between FAM and TAMRA became closer. The change in FRET with the addition of K^+^ increased as the distance between the FAM and TAMRA of the PSO derivative became closer. This is likely due to the increase in FRET efficiency as the distance between PSOs approaches. The FRET efficiency seems to have improved because the delta ratio increases as the FAM and TANRA distances become closer.

PSO **6** and **7** have a flexible GGS linker in the peptide portion. Comparison of **7** and **8**, which have the same peptide length but different peptide sequences, shows that the difference in peptide sequence has little effect on the affinity for K^+^ and the selectivity of K^+^ for Na^+^ in the presence of excess Na^+^. Comparison of **1**, **8**, and **9** shows that the distance between biotin and TAMRA has little effect on the affinity for K^+^ or Na^+^. Focusing on the length of oligonucleotides, **1** and **2**, which have the same peptide sequence, **2** without thymine linker has a higher affinity for K^+^ and a larger ΔRatio, indicating that FRET efficiency is improved.

Similar behavior was observed for **10**–**12** with (SGG)_5_KS linkers and different numbers of thymines. These results indicate that **5** has high potential as a PSO because of its high affinity for K^+^ and large ΔRatio of FAM decrease and TAMRA increase. These results indicate that **5** has high potential as a PSO because of the large decrease in FAM fluorescence and increase in TAMRA fluorescence, the large ΔRatio, the small *K*_d_ relative to K^+^ in the presence of 145 mM NaCl, and the high selectivity of K^+^ over Na^+^.

The behavior of PSO **5** was checked in the presence of StAv in more detail. The *K*_d_ obtained from the fluorescence change when K^+^ was added to 0.2 μM **5**, 0.3 μM StAv was 17.9 ± 1.5 mM, ΔRatio was 0.78, and ΔRatio decreased significantly with the addition of StAv. However, in the presence of 1 μM B-NES, the *K*_d_ to KCl was 4.6 ± 0.7 mM and ΔRatio was 2.1, indicating improved affinity to KCl and recovery of ΔRatio. *K*_d_ relative to NaCl was also 634 ± 40.9 mM, with the highest K^+^/Na^+^ of 396 times. The dissociation constants (or ΔRatio), when one of the four biotin-binding sites of StAv was bound by **5** and the remaining, three by biotin, or when all four binding sites of StAv were bound by **5**, were 12.4 ± 1.2 mM (1.0) and 14.4 ± 2.4 mM (1.3). This result suggests that the binding of a biotin-linked peptide to the biotin-binding site of StAv is more efficient than the binding of biotin alone to the biotin-binding site of StAv, which may have resulted in an increase in FRET efficiency.

### 3.4 Method of Extracellular Immobilization of PSO

Two methods of extracellular immobilization of PSO were investigated.

**FIGURE 4:**
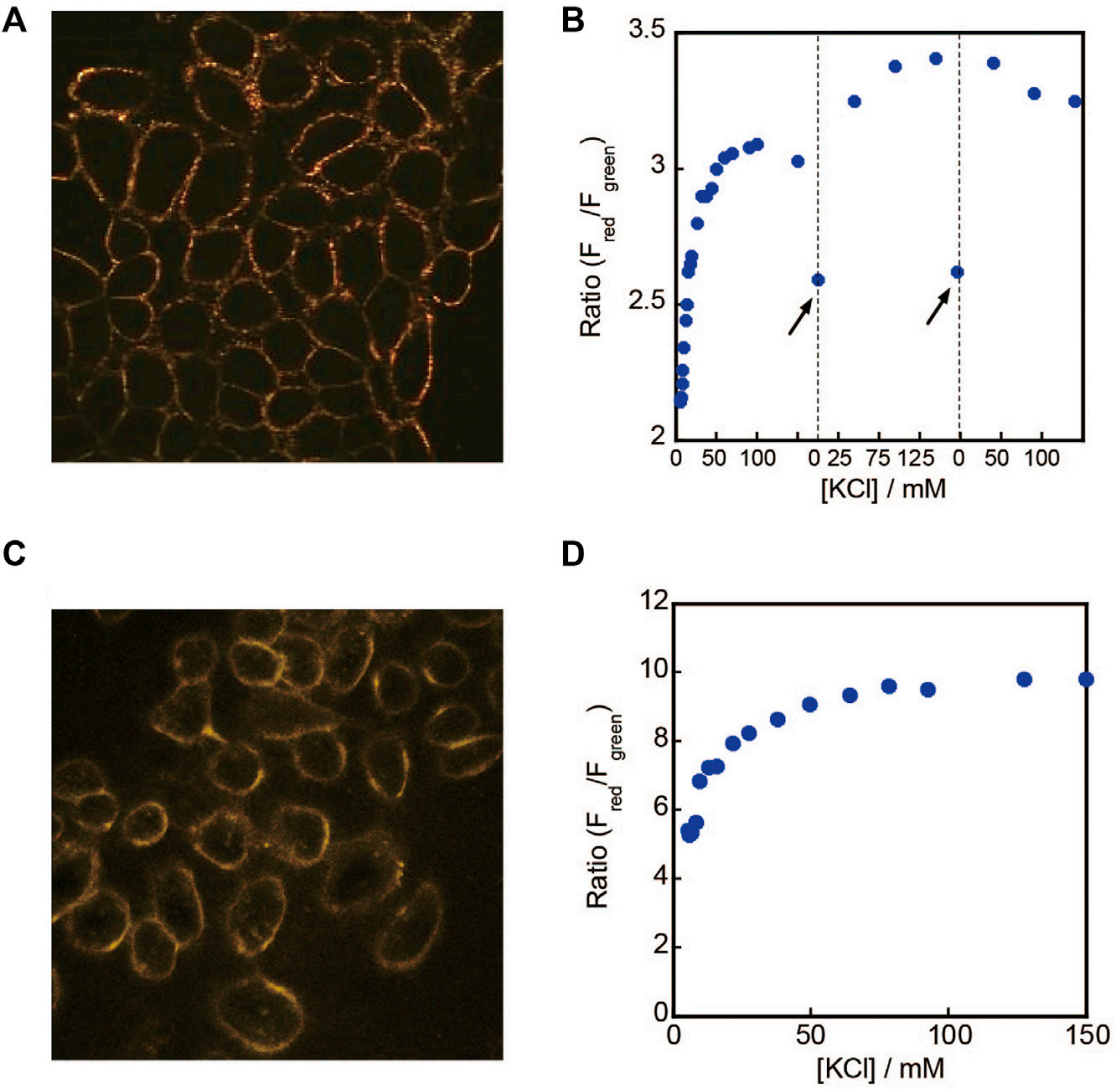
**(A)** Extracell imaging by **1**-StAv-ConA, **(B)** fluorescence ratio of F_red/_F_green_ (F_red_: F.I. of 550–640 nm,/F_green_: F.I. of 495–540 nm) after adding KCl, *λ*_ex_ = 488 nm. **(C)** Extracell imaging by **1**-StAv-sulfo-NHS-biotin, **(D)** fluorescence ratio of F_red/_F_green_ after adding KCl, *λ*_ex_ = 488 nm. (↑) indicates the change of DMEM medium.

First, biotinylated concanavalin A (biotinylated ConA), which binds to cell surface sugars, was used, and 0.5 μM biotinylated ConA, 5.0 μM StAv, 5.0 μM **1** were applied in that order to dishes cultured with HeLa cells (Figure 4A). Second, Sulfo-NHS-biotin (Segu et al., 2011), which binds to amino groups on the cell surface, was used, and dishes containing HeLa cells were treated with 0.5 mg/ml Sulfo-NHS-biotin, 5.0 μM StAv, and 5.0 μM **1** in that order (Figure 4C). Cells thus PSO-displayed were observed by confocal microscopy. Treatment with excess Con A, as well as excess sulfo-NHS-biotin, caused cell shrinkage and migration of **1** into the cells. Therefore, **1** was immobilized for 2 h for Con A and about 1 h for sulfo-NHS-biotin under the conditions shown next, as conditions under which no changes in cell morphology occurred.

The initial KCl concentration of 5.3 mM in the medium allowed for the immobilization of **1** with a *K*_d_ of 21 mM as PSO; when **1** was immobilized via ConA, it was immobilized heterogeneously. On the other hand, when **1** was immobilized via Sulfo-NHS-biotin, **1** was immobilized uniformly. As shown in Figures 4B, D, changes in ratios were observed when KCl was added to the medium. In Figure 4C, when the medium was exchanged, the ratio decreased, indicating that PSO responded to the potassium ion concentration. From the results of Figures 4B, D, the *K*_d_ to KCl was calculated to be 3.15 ± 0.16 mM for Figure 4B and 4.14 ± 0.16 mM for Figure 4D. As shown in Figure 4B, the ratios changed with K^+^ addition even after two medium changes, suggesting that the **1** response is reusable even after three K^+^ additions. However, as shown in Figure 4B, the baseline of the ratio value shifted after the second and third addition of K^+^, and the *K*_d_ calculated using this ratio change was 1.71 ± 0.15 mM, which was slightly changed compared to the first value. This result may be due to minute changes at the cell surface, since the addition of K^+^ to solution of **1** is reproducible.

### 3.5 Extracellular Imaging With PSO **1**

**FIGURE 5:**
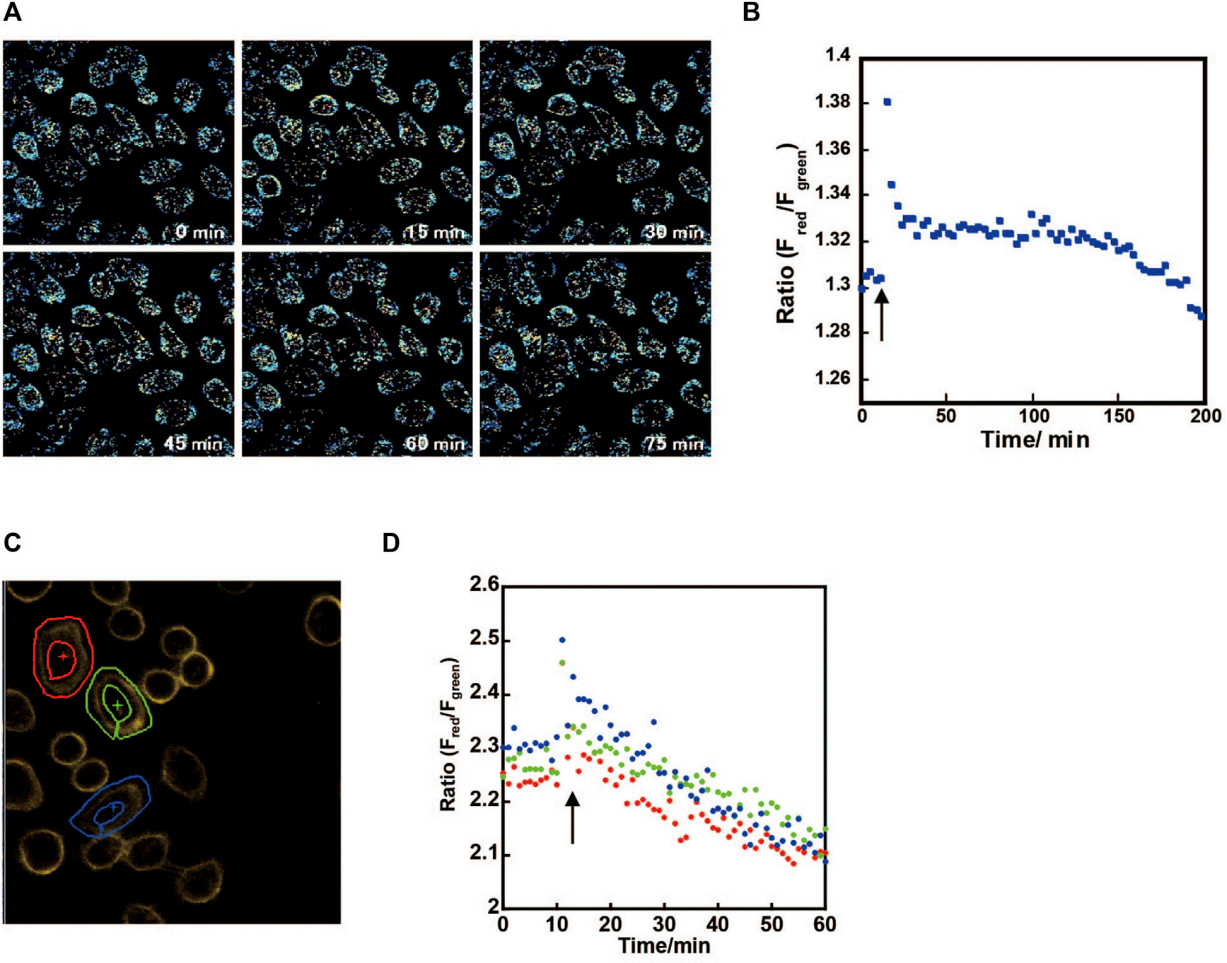
**(A)** Cell surface imaging by **1**-StAv-ConA after adding 10 μM Amphotericin B, 10 μM Bumetanide, and 10 μM Ouabain/DMEM at 11 min (↑), **(B)** fluorescence ratio of F_red/_F_green_ (F_red_: F.I. of 575–595 nm,/F_green_: F.I. of 508–528 nm), *λ*_ex_ = 488 nm in all cells. **(C)** Extracell imaging by **1**-StAv-sulfo-NHS-biotin and **(D)** fluorescence ratio of F_red/_F_green_ (F_red_: F.I. of 554–620 nm, F_green_: F.I. of 500–554 nm), *λ*_ex_ = 488 nm after adding 26 μM Amphotericin B at 11 min (↑). The three cells shown in **(C)** were surrounded by ROI (region of interests), and their fluorescence ratios were shown in **(D)**.

The ratios were calculated from fluorescence imaging images of HeLa cells immobilized with ConA, StAv, and **1** in turn and 10 μM Amphotericin B, 10 μM Bumetanide, and 10 μM Ouabain as apoptosis inducers (Figures 5A,B). Outside the cells, an increase in ratio, i.e., an increase in KCl concentration, was observed immediately after the reagent was added at 11 min. In the intracellular imaging previously reported, a decrease in the ratio, i.e. a decrease in KCl concentration, was also observed immediately after the addition of the apoptosis-inducing agent. In extracellular imaging, the ratio decreased over 30 min and then remained constant. Since the imaging was performed at the cell surface, it is likely that the rate of KCl efflux was constant after 30 min of addition and no change in the ratio occurred. The ratio decreased after 120 min, but the DIC images showed that the cells had developed shrinkage, indicating that cell death had occurred. As a negative control, the cell morphology and ratio did not change after 200 min of observation, indicating that ConA can be used to display cells for about 200 min. When HeLa cells were immobilized with Sulfo-NHS-biotin, StAv, and **1** in turn, and only Amphotericin B was added 11 min after the start of monitoring, a similar increase in ratio was observed immediately after addition, and a gradual decrease in ratio was observed over 60 min (Figures 5C,D). Adding only Amphotericin B did not induce apoptosis and allowed us to monitor the gradual efflux of KCl. In Figures 5B,D, slow decrease in the ratio of **1** was observed after the rapid change in the ratio corresponding to the rapid K^+^ efflux due to the addition of apoptosis inducers. In addition, as shown in Figure 4B, a corresponding change in ratio of **1** was observed when the K^+^ concentration in the medium changed due to medium replacement. These results indicate that **1** has a reusable response to K^+^.

## 4 Conclusion

The *K*_d_ to KCl was calculated when the peptide and oligonucleotide sequences of PSO, a peptide–oligonucleotide conjugate, were changed. All sequences showed high K^+^ selectivity, but the difference in fluorescence ratios was greatest when the distance between the 5′ end of the peptide chain, especially the oligonucleotide, and FAM was short, and also when the TBA sequence did not contain a linker. It was found that FRET efficiency was better when the distance between FRET pairs was as close as possible. We also expected that closer FAM and TAMRA would affect the folding of K^+^ ions in TBA, but the range was about 5–20 mM for *K*_d_. As for KCl imaging, since the extracellular KCl concentration is approximately 5 mM, **1** with a *K*_d_ of about 20 mM, as shown in Figure 5, it would be suitable to monitor KCl efflux from inside the cell where there is about 140 mM KCl, and to see changes in intercellular signaling such as neurons. In this case, imaging with probes with lower *K*_d_, such as **6**, **8**, and **13**, would be suitable. The library shown here should serve as a reference for imaging KCl for different purposes.

## Data Availability

The datasets presented in this study can be found in online repositories. The names of the repository/repositories and accession number(s) can be found in the article/Supplementary Material.

## Abbreviations

ConA, concanavalin A; FAM, fluorescein; FRET, Förster resonance energy transfer; ROI, region of interests; StAv, streptavidin; TBA, thrombin-binding aptamer; TAMRA, tetramethylrhodamine.
