# Supplementary material for: Fluorescence Imaging of Extracellular Potassium Ion Using Potassium Sensing Oligonucleotide
Source: Front Chem. 2022 Jul 8;10:922094. doi: 10.3389/fchem.2022.922094 (PMC9306769; doi:10.3389/fchem.2022.922094)
Supplement: Supplementary file 1 [file DataSheet1.PDF]

Supplementary Material

1. Materials

Reagents for peptide synthesis (Supplementary Table S1) were purchased from Watanabe Chemical Industries Ltd. (Hiroshima, Japan). Peptide was synthesized by PSSM-8 (Shimadzu Corp. Kyoto, Japan). B-NES was synthesized according to the previously report (Ohtsuka et al., 2012). Matrix-assisted laser desorption ionization-TOF MS (MALDI-TOF MS) was measured by Microflex (Bruker Co., Billerica, MS) with α-cyano-4-hydroxycinnamic acid (α-CHCA) or 3-hydroxy propionic acid (3-HPA) as a matrix for peptide or oligonucleotide, respectively. Analysis and purification by reversed phase HPLC were carried out using a Waters e2695 separations Modules, Waters 2998 Photodiode Array Detector, and Waters 2475 Multi λ Fluorescence Detector. Inertsil ODS-3 column (4.6×250 mm, GL Science Inc., Tokyo, Japan) or Mightysil RP-18 column (4.6×250 mm) (Kanto Chemical Co., Inc., Japan) were used for peptide or oligonucleotide separation, respectively.

Table S2. Reagents for peptide synthesis used in this experiment

Amino acid	Amino acid unit for Fmoc chemistry	Mwt	165 μmol equivalent / mg
cysteine (C)	Fmoc-Cys(Trt)-OH	585.72	96.7
glycine (G)	Fmoc-Gly-OH	297.31	49.1
serine (S)	Fmoc-Ser(tBu)-OH	383.44	63.3
lysine (K)	Fmoc-Lys(Boc)-OH	468.55	77.3
Acp*	Fmoc-Acp(6)-OH	353.42	58.3
Biotin	Biotin-OH	244.31	40.3

* 6-((((9*H*-Fluoren-9-yl)methoxy)carbonyl)amino)hexanoic acid

2 Synthesis of 2-12

2 - **12** were synthesized according to the scheme S1.

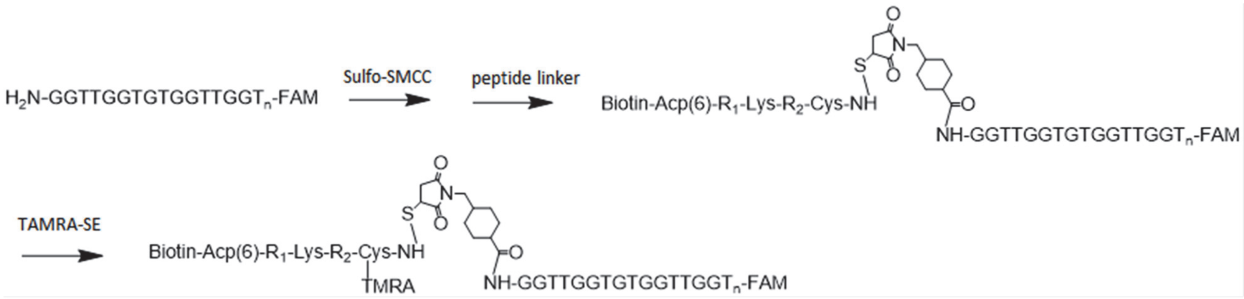

	R1	R2	n
2	GGGGG	GGGGG	0
3	GGGGG	GGG	0
4	GGGGG	G	0
5	GGGGG	-	0
6	GGSGG	GGSGG	2
7	SGG	SGG	2
8	GGG	GGG	2
9	GGG	GGGGG	2
10	(SGG)5	S	0
11	(SGG)5	S	1
12	(SGG)5	S	2

Scheme S1.

2.1 Synthesis of peptide linker

Biotin-Acp-G_5KG_5C-NH_2, Biotin-Acp-G_5KG_3C-NH_2, Biotin-Acp-G_5KGC-NH_2, Biotin-Acp-G_5KC-NH_2, Biotin-Acp- $G_2SG_2KG_2SG_2C$-NH_2, Biotin-Acp-SG_2KSG_2C-NH_2, Biotin-Acp-G_3KG_3C-NH_2, Biotin-Acp- G_3KG_5C-NH_2, and Biotin-Acp-$(SGG)_5KSC$-NH_2 as peptide linker for 2-12, respectively, are biotinylated at the N-terminus and cysteine at the C-terminus. The peptide synthesis for 2-12 was carried out using the peptide synthesizer (PSSM-8) with 30 mg of Fmoc-NH-SAL resin (0.55 mmol/g, Watanabe Chemical) and Fmoc amino acid (Supplementary Table 1, 165 μmol each). After peptide synthesis, the obtained resin was dissolved in 400 μL of the solution containing 370 μL trifluoroacetic acid (TFA), 10 μL water, 10 μL triisopropylsilane, and 10 μL ethanedithiol and kept for 3 h at room temperature to cleave the synthesized peptide from the resin and remove the protection group. The precipitate was obtained after addition of 5 mL of diethyl ether with the filtrate. The precipitate was collected by centrifugation (5000 rpm, 1 min, 4 ºC), and washed with the following solvent order: 5 mL of diethyl ether, diethyl ether, ethyl acetate, and diethyl ether. The obtained white powder was collected by centrifugation and dried under reduced pressure for 2 h. The peptide was purified by the reversed phase HPLC, Waters e2695 separations Modules with the Inertsil ODS-3 column under gradient elution from 0 % to 100 % of 70% acetonitrile in water containing 0.1 % TFA, at the flow rate of 1 mL/min (Figure S1). The elution was monitored at 210 nm and a fraction containing target peptide was collected and lyophilized. MALDI-TOF mass spectrum of the obtained white powder was measured with α-CHCA as a matrix. MALDI-TOF-MS (positive mode, α-CHCA) m/z (Biotin-Acp-G_5KG_5C-NH_2) = 1159.90 (calculated value of $C_{45}H_{75}O_{15}N_{17}S_2+H^+$ = 1159.32), m/z (Biotin-Acp-G_5KG_3C-NH_2) = 1044.24 (calculated value of $C_{41}H_{69}O_{13}N_{15}S_2+H^+$ = 1045.22), m/z (Biotin-Acp-G_5KGC-NH_2) = 930.13 (calculated value of $C_{37}H_{63}O_{11}N_{13}S_2+H^+$ = 931.12), m/z (Biotin-Acp-G_5KC-NH_2) = 873.08 (calculated value of $C_{35}H_{60}O_{10}N_{12}S_2+H^+$ = 874.07), m/z (Biotin-Acp- $G_2SG_2KG_2SG_2C$-NH_2) = 1219.64 (calculated value of $C_{47}H_{80}N_{18}O_{16}S_2+H^+$ = 1218.39), m/z (Biotin-Acp-SG_2KSG_2C-NH_2) = 991.12 (calculated value of $C_{39}H_{67}N_{13}O_{13}S_2+H^+$ = 991.17), m/z (Biotin- Biotin-Acp-G_3KG_3C-NH_2) = 930.92 (calculated value of $C_{37}H_{63}O_{11}N_{13}S_2+H^+$ = 931.12), m/z (Biotin-Acp- G_3KG_5C-NH_{22}) = 1045.24 (calculated value of $C_{41}H_{69}O_{13}N_{15}S_2+H^+$ = 1045.22), m/z (Biotin-Acp-$(SGG)_5KSC$-NH_2) = 1680.43 (calculated value of $C_{63}H_{105}N_{23}O_{27}S_2+H^+$ = 1681.79).

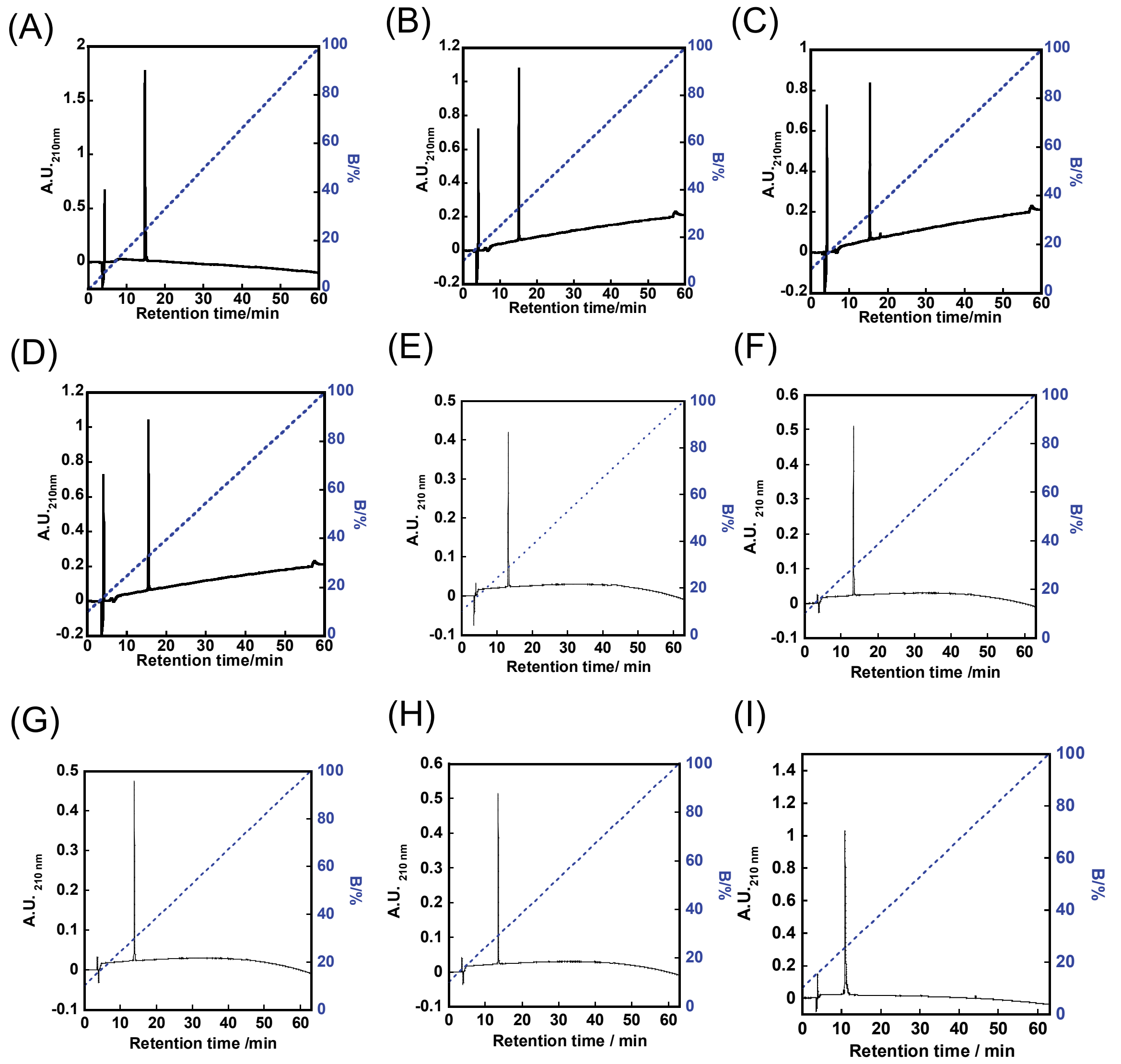

Figure S1. RP-HPLC of synthesized peptide units. (A) Biotin-acp-G_5KG_5C for **2**, (B) Biotin-acp-G_5KG_3C for **3**, (C) Biotin-acp-G_5KGC for **4**, (D) Biotin-acp- G_5KC for **5**, (E) Biotin-acp-$G_2SG_2KG_2SG_2C$ for **6**, (F) Biotin-acp-SG_2KSG_2C for **7**, (G) Biotin-acp-G_3KG_3C for **38**, (H) Biotin-acp-G_3KG_5C for **9**, and (I) Biotin-acp- $(SGG)_5KSC$ for **10-12**.

1.2 Linkage reaction between the linker peptide and the modified oligonucleotide

Biotin-acp-G_5KG_5C-5'-GGTTGGTGTGGTTGG-3'-FAM, Biotin-acp-G_5KG_3C-5'-GGTTGGTGTGGTTGG-3'-FAM, Biotin-acp-G_5KGC-5'-GGTTGGTGTGGTTGG-3'-FAM, Biotin-acp-G_5KC-5'-GGTTGGTGTGGTTGG-3'-FAM, Biotin-acp-$G_2SG_2KG_2SG_2C$-5'-GGTTGGTGTGGTTGGTT-3'-FAM, Biotin-acp-SG_2KSG_2C-5'-GGTTGGTGTGGTTGGTT-3'-FAM, Biotin-acp-G_3KG_3C-5'-GGTTGGTGTGGTTGGTT-3'-FAM, Biotin-acp-G_3KG_5C-5'-GGTTGGTGTGGTTGGTT-3'-FAM, Biotin-acp-$(SGG)_5KSC$-5'-GGTTGGTGTGGTTGG-3'-FAM, Biotin-acp-$(SGG)_5KSC$-5'-GGTTGGTGTGGTTGGT-3'-FAM, Biotin-acp-$(SGG)_5KSC$-5'-GGTTGGTGTGGTTGGTT-3'-FAM (Underlined parts are single letter designations of peptides) as peptide-oligonucleotide conjugate for 2 – 12, respectively, were synthesized as follows. The corresponding 15, 16, or 17-meric oligonucleotide (12 nmol) containing thrombin binding aptamer sequence carrying amino moiety and FAM at 5'- and 3'-termini, respectively, (NH_2-5'-GGTTGGTGTGGTTGG-3'-FAM, NH_2-5'-GGTTGGTGTGGTTGGT-3'-FAM, NH_2-5'-GGTTGGTGTGGTTGGTT-3'-FAM) was custom synthesized by Sigma-Genosys (Hokkaido, Japan) and dissolved in 24 μL of 1×PBS buffer (pH 7.4) and 1 mg of sulfo-SMCC (sulfosuccinimidyl-4-(N-maleimidomethyl)-cyclohexane-1-carboxylate, Thermo Fisher Scientific) and 20 μL of DMSO was added to this solution and mixed with a Vortex mixer for 1.5 h. Five hundred μL of water was added to the oligonucleotide, and purified with NAP-5 column (GE Healthcare Life Science, Japan) by elution with water and lyophilized. The obtained powder (ca. 12 nmol) and peptide linker 1.2 mg was dissolved in 100 μL of 1×PBS buffer (pH 7.4) and mixed with a vortex mixer for 3 h by shading. The peptide oligonucleotide conjugate was purified by the reversed phase HPLC, Waters e2695 separations Modules with the Mightysil RP-18 column under gradient elution from 0 % to 100 % of 10% CH_3CN in 0.1 M TEAA (pH 7.0) and 70% CH_3CN in 0.1 M TEAA (pH 7.0) at the flow rate of 1 mL/min (Figure S2). The elution was monitored at 260, 495 nm by Waters 2998 Photodiode Array Detector and a fraction containing peptide oligonucleotide conjugate was collected and lyophilized.

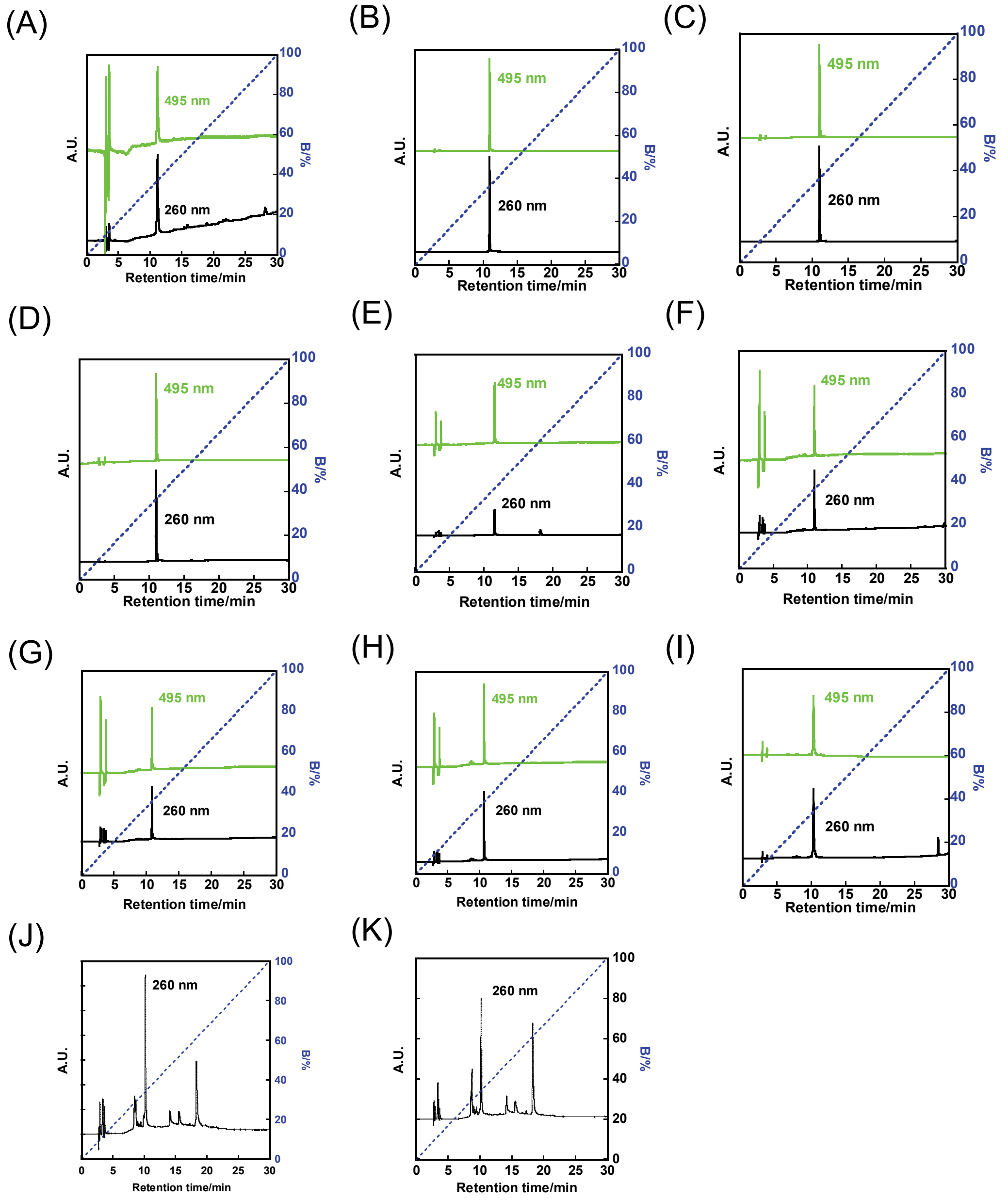

Figure S2. RP-HPLC of the peptide-oligonucleotide conjugates in this experiment. (A) Biotin-acp-G$_5$KG$_5$C-5'-GGTTGGTGTGGTTGG-3'-FAM for **2**, (B) Biotin-acp-G$_5$KG$_3$C-5'-GGTTGGTGTGGTTGG-3'-FAM for **3**, (C) Biotin-acp-G$_5$KGC-5'-GGTTGGTGTGGTTGG-3'-FAM for **4**, (D) Biotin-acp-G$_5$KC-5'-GGTTGGTGTGGTTGG-3'-FAM for **5**, (E) Biotin-acp-G$_2$SG$_2$KG$_2$SG$_2$C-5'-GGTTGGTGTGGTTGGTT-3'-FAM for **6**, (F) Biotin-acp-SG$_2$KSG$_2$C-5'-GGTTGGTGTGGTTGGTT-3'-FAM for **7**, (G) Biotin-acp-G$_3$KG$_3$C-5'-GGTTGGTGTGGTTGGTT-3'-FAM for **8**, (H) Biotin-acp-G$_3$KG$_5$C-5'-GGTTGGTGTGGTTGGTT-3'-FAM for **9**, (I) Biotin-acp-(SGG)$_5$KSC-5'-GGTTGGTGTGGTTGG-3'-FAM for **10,** (J) Biotin-acp-(SGG)$_5$KSC-5'-GGTTGGTGTGGTTGGT-3'-FAM for **11**. (K) 11; Biotin-acp-(SGG)$_5$KSC-5'-GGTTGGTGTGGTTGGT-3'-FAM. Underlined parts are single letter designations of peptides.

1.3 Addition of TAMRA to peptide oligonucleotide conjugate

The peptide oligonucleotide conjugates, 2-12 were synthesized as follows. The peptide oligonucleotide conjugates was dissolved in 100 μL of MiiliQ, 10 μL of 3 M KCl, and 30 μL of 100 mM 2-[4-(2-hydroxyethyl)piperazin-1-yl] ethanesulfonic acid (HEPES) NaOH (pH 8.0), and 33 μM 5-carboxytetramethylrhodamine (TAMRA) NHS ester (Biotech) in DMSO 30 μL was added to this solution and mixed with a Vortex mixer for 4 h at r.t by shading. After reaction, excess TAMRA was removed with NAP-5 NAP-5 column by elution with water and lyophilized. The target peptide oligonucleotide conjugate was purified by the reversed phase HPLC, Waters e2695 separations Modules with the Mightysil RP-18 column under gradient elution from 0 % to 100 % of 10% CH_3CN in 0.1 M TEAA (pH 7.0) and 70% CH_3CN in 0.1 M TEAA (pH 7.0) at the flow rate of 1 mL/min (Supplementary Figure 3). The elution was monitored at 260, 495, 560 nm by Waters 2998 Photodiode Array Detector and a fraction containing peptide oligonucleotide conjugate was collected and lyophilized. MALDI-TOF mass spectrum of the obtained white powder was measured with 3-HPA as a matrix. MALDI-TOF-MS (positive mode, 3-HPA) m/z (2) 7343.7 (calculated value of $C_{269}H_{339}O_{129}N_{80}S_2P_{15}$-$H^-$ = 7958.38), m/z (3) 7233.9 (calculated value of $C_{265}H_{333}O_{127}N_{78}S_2P_{15}$-$H^-$ = 7171.87), m/z (4) 7121.8 (calculated value of $C_{261}H_{327}O_{125}N_{76}S_2P_{15}$-$H^-$ = 7057.76), m/z (5) 7061.7 (calculated value of $C_{259}H_{324}O_{124}N_{75}S_2P_{15}$-$H^-$ = 7067.55), m/z (6) 8032.6 (calculated value of $C_{291}H_{369}O_{149}N_{84}S_2P_{17}$-$H^-$ = 8081.55), m/z (7) 7797.9 (calculated value of $C_{283}H_{357}O_{145}N_{80}S_2P_{17}$-$H^-$ = 7790.34), m/z (8) 7736.6 (calculated value of $C_{281}H_{353}O_{143}N_{80}S_2P_{17}$-$H^-$ = 7730.29), m/z (9) 7854.3 (calculated value of $C_{285}H_{359}O_{145}N_{82}S_2P_{17}$-$H^-$ = 7844.39), m/z (10) 7824.7 (calculated value of $C_{287}H_{369}O_{141}N_{86}S_2P_{15}$-$H^-$ = 7874.91) , m/z (11) 8190.9 (calculated value of $C_{297}H_{382}O_{150}N_{88}S_2P_{16}$-$H^-$ = 8177.940), , m/z (12) = 8072.66 (calculated value of $C_{307}H_{395}O_{159}N_{90}S_2P_{17}$-$H^-$ = 8480.97).

Yield: **2**: 14% (1.43 nmol), **3**: 6% (0.60 nmol), **4**: 17% (1.7 nmol), **5**: 19% (1.9 nmol, **6**: 10% (0.7 nmol), **7**: 15% (1.1 nmol), **8v** 14% (1.0 nmol), **9**: 11% (0.8 nmol), **10**: 6% (0.53 nmol), **11**: 13% (1.1 nmol), **12**: 27% (1.4 nmol)

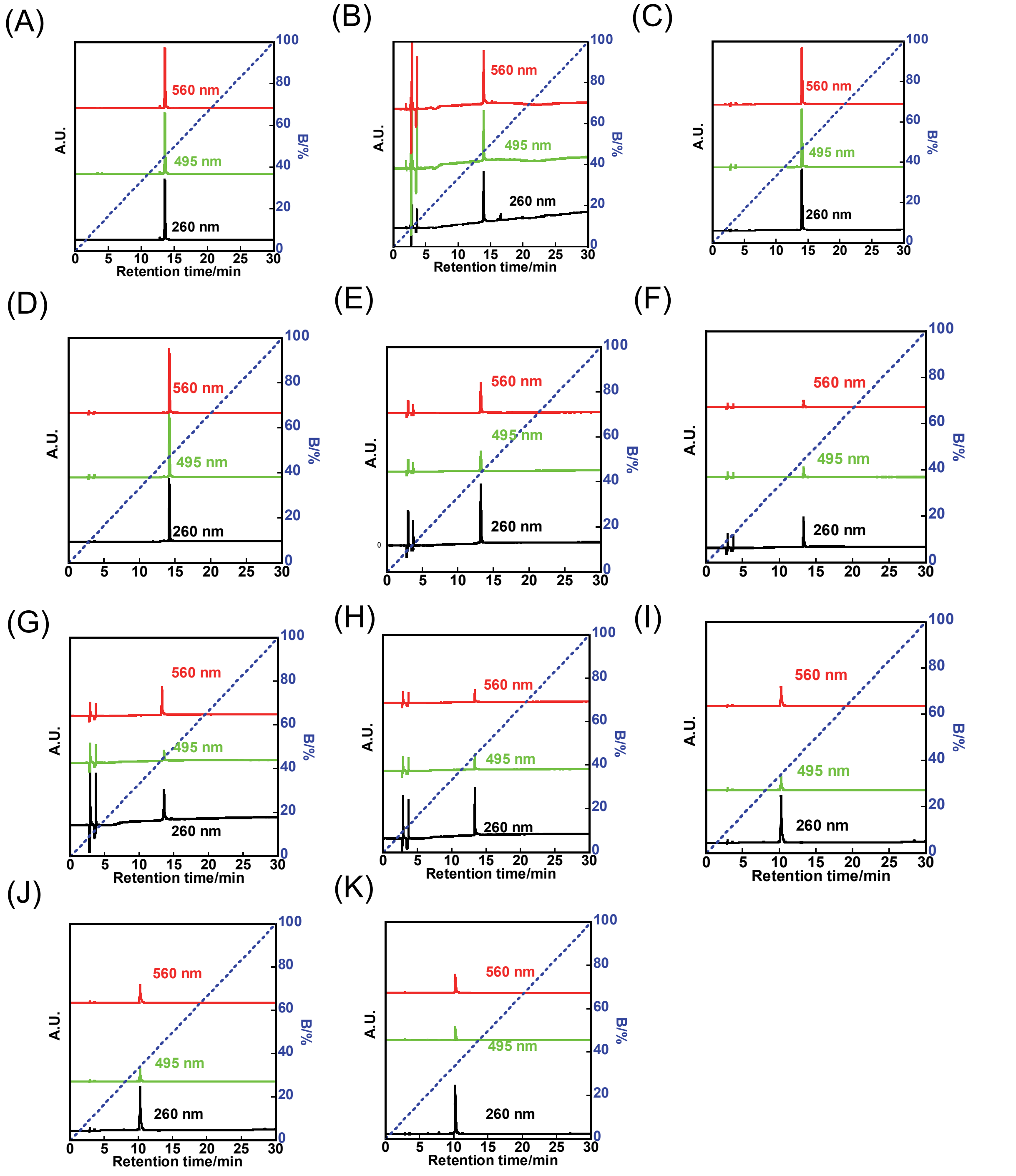

Figure S3. RP-HPLC of **2** (A), **3** (B), b (C), **5** (D), **6** (E), **7** (F), **8** (G), **9** (H), **10** (I), **11** (J), **12** (K).
